# OSSE – open source registry software solution

**DOI:** 10.1186/1750-1172-9-S1-O9

**Published:** 2014-11-11

**Authors:** Marita Muscholl, Martin Lablans, Thomas OF Wagner, Frank Ückert

**Affiliations:** 1 University Medical Center, Mainz, Germany; 2 University Hospital, Frankfurt, Germany

## Project goal

*OSSE* (*Open Source-Registersystem für Seltene Erkrankungen in der EU / Open Source Registry System for Rare Diseases in the EU*) provides patient organizations, physicians and scientists with open-source software for the creation of patient registries. As a result, the national registry landscape is improved to comply with European principles regarding e.g. minimum data set and data quality, as summarized in the EUCERD recommendation on rare disease registries. Also, the necessary interoperability is achieved to facilitate federation of those registries on a national and international level.

## Overall concept

### The registry toolbox

*OSSE* primarily provides a registry toolbox that allows for the definition of forms for longitudinal and medical core data and of the corresponding data schema by means of a registry editor. Common data elements, which can be chosen to build the forms, are defined within a metadata repository (MDR) following ISO/IEC 11179. This approach provides semantic interoperability and data quality. All harmonized data sets for rare diseases will be available through the MDR. Further items can be added at any time while building disease-specific registries and will be available for all *OSSE*-compliant registries. The *OSSE* registry provides plausibility checks regarding ranges defined in the MDR and field-specific dependencies, data versioning, workflow support, interfaces for configurable data import and export, and role-based access control.

### Pseudonymization

To meet data protection requirements, *OSSE* supports pseudonymization of patient data: the part of the data allowing for the identification of patients is replaced by a pseudonym and stored separately in the patient list that should be controlled by a trusted third party. For pseudonymization we use the opensource software “Mainzelliste” ["http://www.mainzelliste.de"].

### Distributed search

Interoperability between different *OSSE* registries is achieved by a distributed search infrastructure taking into account data ownership and privacy aspects. The central *search broker* allows specified search queries in all *OSSE*-compliant registries based on the existing MDR items. Results are presented to the person in charge of the data management for each registry; the exchange or dissemination of data remains in full control of the data owner.

